# Level of implementation of multimodal strategies for infection prevention and control interventions and prevalence of healthcare-associated infections in Northern Italy

**DOI:** 10.1186/s13756-024-01398-1

**Published:** 2024-04-11

**Authors:** Costanza Vicentini, Roberta Bussolino, Claudia Gastaldo, Marta Castagnotto, Fortunato “Paolo” D’Ancona, Carla Maria Zotti, on behalf of the Working group “Unità Prevenzione Rischio Infettivo (UPRI), Regione Piemonte”, Fabrizio Bert, Cesare Bolla, Roberta Broda, Francesco D’Aloia, Francesco De Gregorio, Francesco Di Nardo, Piero Fenu, Gerolamo Ferrauto, Mauro Franco, Scipione Gatti, Franca Gremo, Agostino Maiello, Domenica Morabito, Aida Muca, Roberta Palladino, Alessandro Paudice, Paolo Pellegrino, Claudio Plazzotta, Simone Porretto, Giorgio Ripamonti, Maurizio Salvatico, Paola Silvaplana, Carlo Silvestre, Domenico Tangolo, Valentina Venturino, Maria Rita Viroletti

**Affiliations:** 1 Department of Public Health and Pediatrics, University of Turin, Via Santena 5 Bis, 10126 Turin, Italy; 2 Epidemiology, Biostatistics and Mathematical Modeling Unit (EPI), Department of Infectious Diseases, Istituto Superiore Di Sanità (ISS), Viale Regina Elena 299, 00161 Rome, Italy

**Keywords:** Healthcare-associated infections, Infection prevention and control, Point prevalence survey, Italy

## Abstract

**Background:**

In November 2022, Italy participated in the third edition of the European Centre for disease prevention and control (ECDC) point prevalence survey (PPS) of healthcare-associated infections (HAIs) in acute-care hospitals. A questionnaire based on the WHO infection prevention and control assessment framework (IPCAF) was included, which aims to investigate multimodal strategies for the implementation of IPC interventions.

**Methods:**

A PPS was conducted using the ECDC PPS protocol version 6.0. The Regional health authority of the region of Piedmont, in north-western Italy, chose to enlist all public acute-care hospitals. Data were collected within one day per each ward, within 3 weeks in each hospital, at hospital, ward and patient level. A score between 0–1 or 0–2 was assigned to each of the 9 items in the IPCAF questionnaire, with 14 points representing the best possible score. HAI prevalence was calculated at the hospital-level as the percentage of patients with at least one HAI over all included patients. Relations between HAI prevalence, IPCAF score, and other hospital-level variables were assessed using Spearman's Rho coefficient.

**Results:**

In total, 42 acute-care hospitals of the region of Piedmont were involved, with a total of 6865 included patients. All participant hospitals reported they employed multimodal strategies to implement IPC interventions. The median IPCAF overall score was 11/14 (interquartile range, IQR: 9.25–12). The multimodal strategy with the highest level of adherence was education and training, followed by communication and reminders. Strategies with the lowest level of adherence were safety climate and culture of change, and system change. Overall HAI prevalence was 8.06%. A weak to moderate inverse relation was found between IPCAF score and HAI prevalence (Spearman’s Rho -0.340, p 0.034). No other significant correlation was found.

**Conclusions:**

This study found a high self-reported overall level of implementation of multimodal strategies for IPC in the region. Results of this study suggest the relevance of the multimodal approach and the validity of the IPCAF score in measuring IPC programs, in terms of effectiveness of preventing HAI transmission.

## Background

Healthcare-associated infections (HAIs) represent a serious public health challenge worldwide with a major impact on patient morbidity, mortality and quality of life [1–3]. Up to 55% of HAIs are estimated to be potentially avoidable through the implementation of appropriate infection prevention and control (IPC) interventions [4].

The World Health Organization (WHO) developed a standardized self-assessment tool, the infection prevention and control assessment framework (IPCAF), which allows to perform a baseline evaluation of IPC practices at the national, sub-national and facility level, and to document and guide improvements over time through repeated measurements [5]. The IPCAF questionnaire assesses eight core elements of IPC: IPC programme; IPC guidelines; IPC education and training; HAI surveillance; multimodal strategies for implementation of IPC activities; IPC practice monitoring and auditing; workload, staffing and employment; built environment, materials and equipment for IPC at facility level. In 2018, a multi-country, cross-sectional study was conducted through semi-structured interviews based on the IPCAF, which revealed that only 12.5% ​​of the 88 participating countries implemented elements of all core components [6].

Since 2011, the European Centre for disease prevention and control (ECDC) has been promoting point prevalence surveys (PPS) of HAIs in acute-care hospitals every five years. According to the latest ECDC PPS surveillance report (based on data collected in 2016–2017), HAI prevalence was 5.9% at the European level [7]. In Italy, HAI prevalence was 8.03% in 2016, corresponding to an estimated annual burden of over 700 disability-adjusted life years (DALYs) per 100.000 general population [1, 7].

In November 2022, Italy participated in the third edition of the ECDC PPS. Among other updates, the PPS3 protocol revised the section addressing IPC practices by including the IPCAF questionnaire, with the option of completing the full questionnaire or a summary version [8]. In Piedmont, a Northern Italian region, 42 acute-care hospitals participated in the PPS3 survey. The Italian national coordinating team chose to use the short version of the IPCAF questionnaire, which aims to investigate multimodal strategies for the implementation of IPC interventions (WHO Core component 5), as proposed by the ECDC [9].

The objective of this study was to assess if a score based on the core items for IPC assessed through the short version of the IPCAF questionnaire correlated with HAI prevalence at the hospital-level.

## Methods

### Study design, protocol and definitions

A national PPS was conducted in November 2022. The ECDC proposed 3 periods for conducting the PPS: April-June 2022, September–November 2022 and April-June 2023; the Italian study was conducted within the second window. The Department of Public Health and Pediatrics of the University of Turin acted as national coordinating center.

An adapted version of the ECDC PPS protocol version 6.0 was used [8, 9]. The PPS protocol adopts European case definitions for HAIs (as proposed by Hospitals in Europe Link for Infection Control through Surveillance, HELICS) as well as National Healthcare Safety Network (NHSN) HAI definitions [10].

### Sampling of hospitals

The selection of the national sample took place through convenience sampling, *i.e*. the 21 Italian regions and hospitals were invited to join the surveillance on a voluntary basis. Due to the multi-level governance of the Italian National health system [11], to ensure regional representativeness each region was asked to contribute to the study with a proportionate number of hospitals based on their population, acute-care hospital bed-days and discharges (considering ordinary admissions to acute care facilities). In addition to this minimum limit, which ranged from 1 to 5 hospitals per region, each region could choose to participate to a greater extent [9]. The Regional health authority of Piedmont, in north-western Italy, chose to enlist all public acute-care hospitals. Not-for-profit and private facilities were also invited to participate on a voluntary basis.

### Data collection

PPS data were collected by trained local hospital staff, including doctors, infection control nurses and other nursing staff. Data were collected within one day per each ward, within 3 weeks in each hospital. All patients admitted to the ward before 8 A.M. on the day of the survey and still present at the time of the PPS were included.

As previously described in detail, data were collected at hospital, ward and patient level. [12]. Hospital-level data included hospital characteristics, structure and process indicators pertaining to IPC, as well as the IPCAF questionnaire. As aforementioned, according to the ECDC protocol it was possible to choose whether to complete the IPCAF questionnaire in full or a summary version [8]; at the national level, the latter option was chosen. Patient-level data included demographic and clinical data, such as presence of invasive devices, severity of underlying conditions, antibiotic use, presence of active HAIs. Additional information was collected for patients receiving one or more antimicrobial treatment or in case of active HAIs.

Data were collected using a REDCap-based online platform, which was previously tested through a pilot PPS [13]. The data collection form was designed by the national coordinating team, and developed in collaboration with software engineers. Access to the platform was restricted to authorized users, in compliance with the EU General data protection regulation (GDPR). The national coordinating center provided on-line training on the study design, protocol and definitions, as well as on how to use the online software and data entry form.

The national coordination centre received data between December 2022 and March 2023 and subsequently performed quality assessment, assembly, and analysis.

### Statistical analysis

Descriptive statistics were used to summarize hospital-level data, namely: size, ownership type, level of care provided, full-time equivalent (FTE) doctors and nurses per 1000 beds, proportion of single rooms, diagnostic capacity, alcohol-based handrub consumption per 1000 patient-days (PDs), participation in surveillance networks. Quantitative variables were summarized using median and interquartile ranges (IQRs) due to non-normal distribution (Shapiro–Wilk tests).

A scoring system was developed to summarize responses to the short version of the IPCAF questionnaire. A score between 0–1 or 0–2 was assigned to each of the 9 items in the questionnaire: use of multimodal strategies to implement infection prevention and control (IPC) interventions; level of implementation of system change, education and training, monitoring and feedback, communication and reminders, safety climate and culture of change;

Scores for each hospital were then added up to obtain an overall score ranging from 0 to 14 points, where 14 points represent the best possible score. Full details of the items included in the questionnaire, scoring system, and survey responses are available in Table 1.

**Table 1:** Items included in the short version of the infection prevention and control assessment framework (IPCAF) questionnaire, scoring system, and survey responses (*N* = 42)

Survey item	Score	Results – n (%)
A. Do you use multimodal strategies to implement infection prevention and control (IPC) interventions?		
• No/Unknown	0	0
• Yes	1	42 (100)
B. Do your multimodal strategies include any or all of the following?		
**System change**		
• Element not included in multimodal strategies	0	6 (14.29)
• Interventions to ensure the necessary infrastructure and continuous availability of supplies are in place	1	19 (45.24)
• Interventions to ensure the necessary infrastructure and continuous availability of supplies are in place, further ergonomics and accessibility are addressed (e.g. best placement of central venous catheter set and tray)	2	17 (40.48)
**Education and training**		
• Element not included in multimodal strategies	0	2 (4.76)
• Written information and/or oral instructions and/or e-learning only	1	10 (23.81)
• Additional interactive training sessions (including simulations and/or bedside training)	2	30 (71.43)
**Monitoring and feedback**		
• Element not included in multimodal strategies	0	0
• Monitoring compliance through process and outcome indicators (e.g. hand hygiene audits or catheterization practices)	1	20 (47.62)
• Monitoring compliance and providing timely feedback of monitoring results to healthcare professionals and other key players	2	22 (52.38)
**Communication and reminder**		
• Element not included in multimodal strategies	0	1 (2.38)
• Reminders, posters or other advocacy/awareness-raising tools to promote the intervention	1	15 (35.71)
• Additional initiatives to improve team communication across units and disciplines (e.g. by establishing regular case conferences and feedback rounds)	2	26 (61.91)
**Safety climate and culture of change**		
• Element not included in multimodal strategies	0	9 (21.43)
• Managers/leaders show visible support and act as champions and role models, promoting an adaptive approach aimed at strengthening a culture that supports IPC, patient safety and quality	1	25 (59.52)
• In addition, teams and individuals are empowered so that they perceive ownership of the intervention (e.g. by participatory feedback rounds)	2	8 (19.05)
C. Is a multidisciplinary team used to implement multimodal strategies?		
• No/Unknown	0	2 (4.76)
• Yes	1	40 (95.24)
D. Do you regularly link to colleagues from quality improvement and patient safety to develop and promote multimodal strategies for IPC?		
• No/Unknown	0	9 (21.43)
• Yes	1	33 (78.57)
E. Do these strategies include bundles or checklists?		
• No/Unknown	0	5 (11.91)
• Yes	1	37 (88.1)

HAI prevalence was calculated at the hospital-level as the percentage of patients with at least one HAI over all included patients on the day of the survey. For this study, COVID-19 patients were excluded, due to the possible confounding effect on HAI transmission [14–16].

Relationships between the following variables: HAI prevalence, IPCAF score, FTE infection control personnel per 1000 beds, number of yearly blood cultures, and number of yearly stool tests for *Clostridium difficile* per 1000 PDs, and yearly alcohol-based hand rub consumption (litres per 1000 PDs), were assessed at the facility level using Spearman's Rho coefficient, as the distribution of variables was not normal. All analyses were performed using IBM SPSS, Version 28.0. Armonk, NY: IBM Corp.

## Results

### Hospital-level data

In total, 42 acute-care hospitals of the region of Piedmont were involved, 3 of which were not-for-profit hospitals (7.14%). The median number of beds was 218.5 (IQR 118.5–277.5). Only 4 hospitals exceeded 500 beds. Regarding the level of care provided, the majority of hospitals provided primary-level care (*n* = 19). Full details of characteristics of participating hospitals, IPC structure, resources, and practices are provided in Table 2.

**Table 2:** Characteristics of participating hospitals, infection prevention and control (IPC) structure, resources, and practices

Characteristic	Value
Hospital size (number of beds), n (%)	
< 200	19 (45.23)
200–500	19 (45.23)
≥ 500	4 (9.52)
Ownership, n (%)	
Public	39 (92.86)
Private	0
Not-for-profit	3 (7.14)
Level of care, n (%)	
Basic	9 (21.43)
Primary level	19 (45.23)
Secondary level	8 (19.05)
Specialized	6 (14.29)
IPC structure and resources	
Infection control personnel, median (interquartile range, IQR)	
Full-time equivalent (FTE) doctors per 1000 beds	2.03 (1.21 -2.78)
FTE nurses per 1000 beds	6.71 (5.18 -9.22)
FTE stewardship consultants per 1000 beds	0.15 (0 – 1.96)
Proportion of single rooms (% over all rooms), median (IQR)	16.2 (11–23)
Diagnostic capacity:	
Number of blood cultures/year (per 1000 patient-days, PDs)	60.1 (28.76–83.75) ^ab^
Number of stool tests for *Clostridium difficile*/year (per 1000 PDs)	5.72 (3.54 -8.57) ^ab^
Possibility of requesting exams during the weekend, n (%)	30 (71.43)
IPC practices	
Alcohol-based hand rub consumption/year (litres per 1000 PDs), median (IQR)	24 (17–30)^a^
Participation surveillance networks, n (%)	
Surgical site infections	38 (90.48)
Urinary tract infections	32 (76.19)
*C. difficile* infections	17 (40.48)
Antibiotic resistance	35 (83.33)
Antibiotic use	29 (69.05)
Other	10 (23.81)

^a^ Data referring to the previous year (2021)

^b^ Data available from 40 hospitals

All participant hospitals reported they employed multimodal strategies to implement IPC interventions. The median IPCAF overall score was 11 (IQR: 9.25–12). Scores assigned to each item are reported in Table 1. The multimodal strategy with the highest level of adherence was education and training, with 30 facilities achieving the highest score for this section, followed by communication and reminders [26 facilities], and monitoring and feedback (22 facilities). Strategies with the lowest level of adherence were safety climate and culture of change, with 9 facilities not implementing this element, and system change (6 facilities). A multidisciplinary team was involved in the implementation of multimodal strategies in the majority of hospitals (*n* = 40, 95.24%).

### Patient-level data

**Fig. 1:**
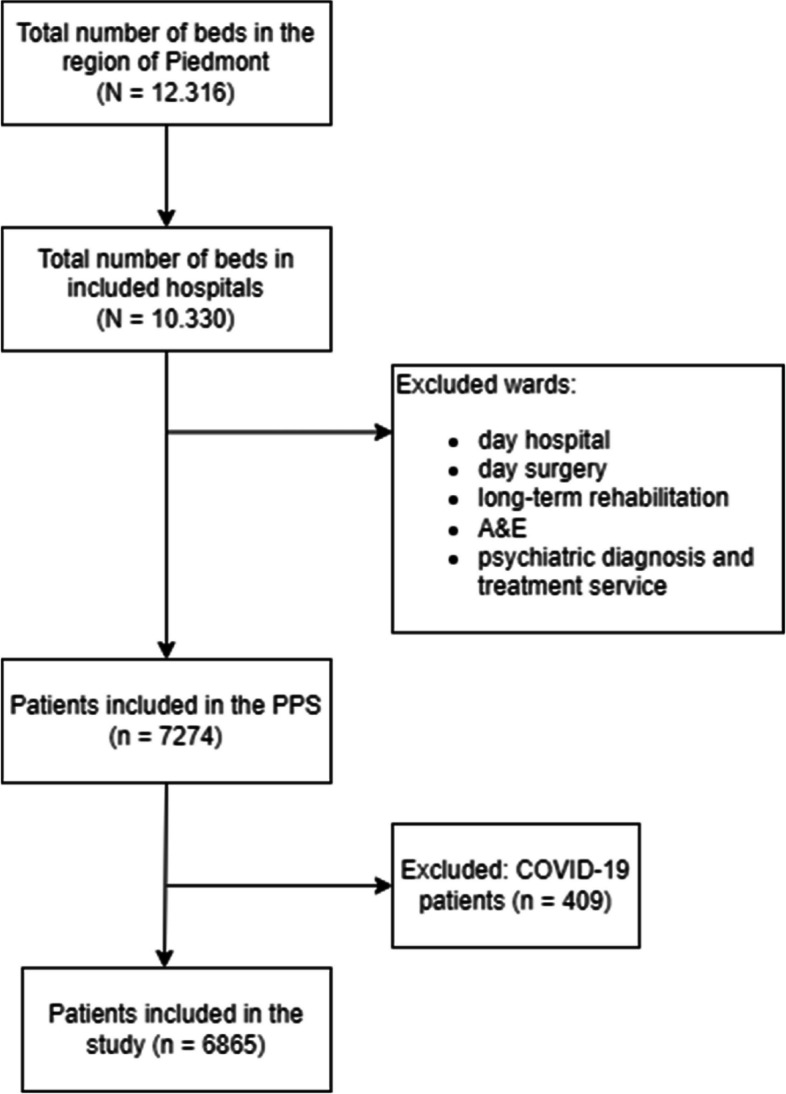
Flow chart of study participants

In total, 7274 patients were included in the PPS, of these, 409 were COVID-19 patients and were therefore excluded from analyses (Fig. 1). Among the remaining 6865 patients, 49.93% were female, and the median age was 72 years (IQR 56–81). The majority of patients were hospitalized in medical wards (40.66%), followed by surgical wards (27.6%).

On the day of the survey, 593 HAIs were recorded among 553 patients (overall HAI prevalence: 8.06%); the mean HAI prevalence among hospitals was 7% (95% confidence interval, CI 5.8%—8.3%). HAIs acquired in the trusts were 81.79% of cases. The 3 most frequently observed HAIs were pneumonia (n = 128, 21.59%), cystitis or other symptomatic lower urinary tract infections (UTIs, n = 125, 21.08%) and bloodstream infections (BSIs, n = 76, 12.82%). Pneumonia was related to patient intubation in 39.1% of cases. Among patients with UTIs, 66.09% had a urinary catheter, and among patients with BSIs, 63.16% had a central venous catheter.

### Correlation analyses

**Fig. 2:**
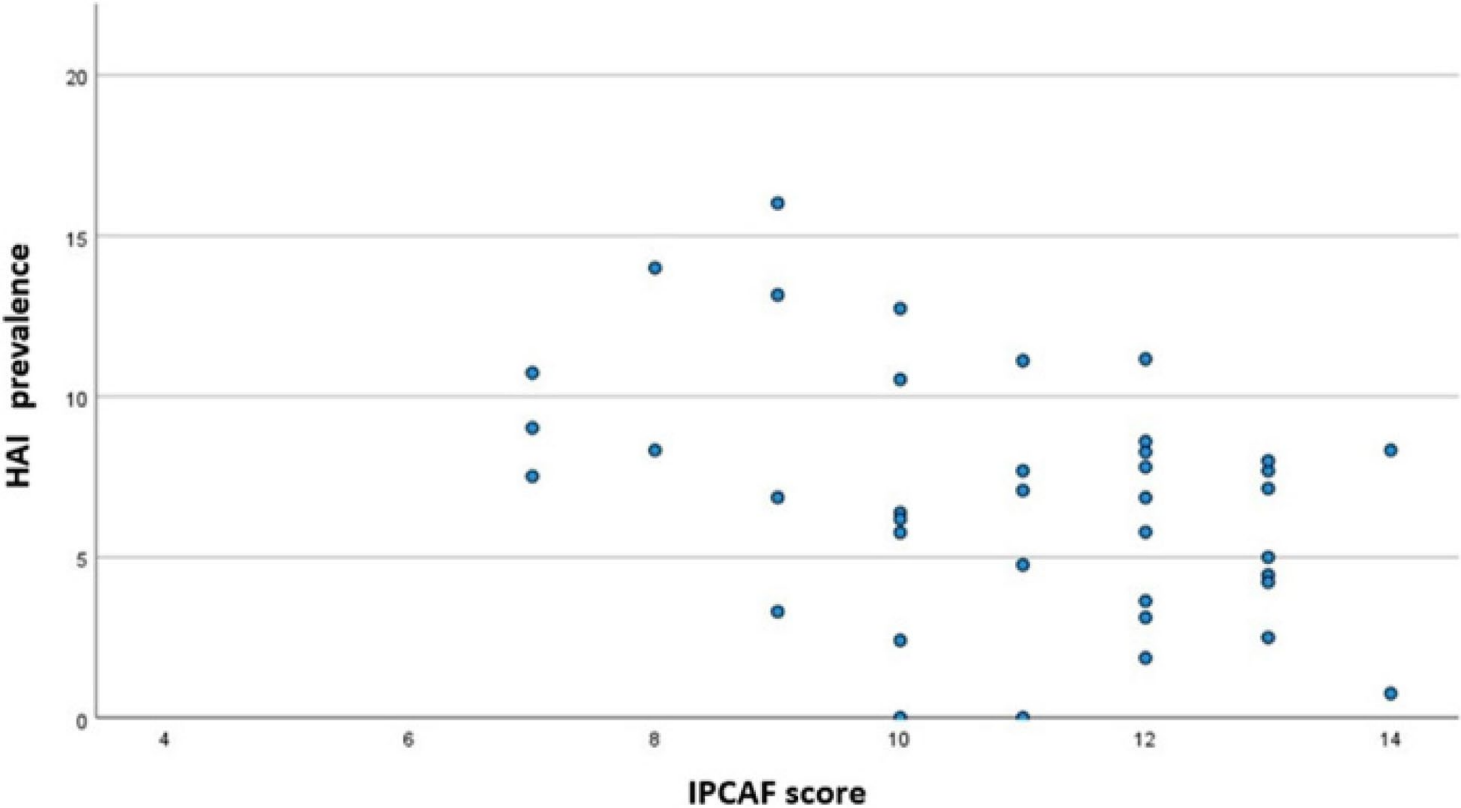
Scatter plot depicting the relation between healthcare-associated infection (HAI) prevalence (%) and infection prevention and control assessment framework (IPCAF) score

As depicted in Fig. 2, a weak to moderate inverse relation was found between IPCAF score and HAI prevalence (Spearman’s Rho -0.340, p 0.034). No other significant correlation was found between HAI prevalence and other considered hospital-level variables, nor between IPCAF score and other considered hospital-level variables.

## Discussion

This study described a region-wide assessment of the level of implementation of multimodal IPC practices among 42 acute-care hospitals of Northern Italy. A standardized questionnaire based on the WHO IPCAF Core component 5 was employed, for which a significant correlation with HAI prevalence was found.

In Italy, antimicrobial resistance (AMR) is a relevant health concern [1, 12]. To address this issue, two Italian National Action Plans to contrast AMR (PNCAR) were issued, in 2017 and 2022 [17, 18]. IPC and HAI surveillance are included among strategic objectives of both plans, and together with appropriate antibiotic use are recognized as the three pillars sustaining the national strategy and coordinated governance of the latest PNCAR.

As the Italian national health system is decentralized, the provision of healthcare within each of the 21 Regions of Italy, including the local implementation of the PNCAR, is consigned to Regional Health Authorities [11]. In the Region of Piedmont, a series of policy measures, quality-driven strategies, and an indicator-based performance evaluation program have been employed to promote surveillance of HAIs and strengthen IPC activities since 2008, within a regional framework. Performance indicators are updated each year by a multidisciplinary working group, and have been targeted towards the objectives outlined in the PNCARs since 2018.

Several aspects of IPC programs have been assessed through the regional indicator system throughout the years, however multimodal strategies, a relatively novel concept, had not yet been systematically investigated. The WHO IPCAF is based on evidence-based guidelines for the implementation of Core components of IPC [19]. Importantly, an inverse correlation between a score based on Core component 5 and HAI prevalence was found in this study, supporting the relevance of the multimodal approach and the validity of the IPCAF score in measuring IPC programs, in terms of effectiveness of preventing HAI transmission.

Our study found a high overall level of implementation of multimodal strategies for IPC (median score 11/14), with the majority of facilities implementing all elements defined by the WHO. Previous evaluations performed in Germany, Austria, and Korea found lower scores for multimodal strategies compared to those assigned to other Core components, leading the Authors to conclude that efforts to strengthen IPC in high-income countries should place further focus on multimodal strategies [20–22].

However, in our Region safety climate and culture of change and system change were identified as areas for improvement. Further efforts should be directed towards empowering teams and individual healthcare professionals so that they perceive ownership of interventions. This issue is particularly relevant in our country, given the lack of accountability and tendency by several stakeholders to avoid taking charge recognized by the ECDC in their country visit to Italy to discuss AMR [23].

According to results of our study, IPC staffing levels are in line with minimum requirements defined by the WHO [24], and the number of IPC doctors and nurses per 1000 beds have slightly increased since the previous edition of the PPS conducted in Piedmont [25]. The majority of hospitals indicated their participation in surveillance networks coordinated within the regional framework: namely surgical site infections, antibiotic resistance, and antibiotic use. A lower degree of participation was found for surveillances outside this system, such as *C. difficile* infections.

Alcohol-based handrub consumption has considerably increased, from a median of 11.1 L per 1000 PDs in 2016 to 24 in the current study [25]. This finding is in line with previous national and international reports of increased consumption of alcohol-based handrub following the COVID-19 pandemic, however efforts should now be dedicated to maintaining high consumption levels beyond the pandemic context [26, 27]. Interestingly, a multimodal approach to promoting hand hygiene practices was adopted by our region, in line with WHO guidelines and through the Hand hygiene self-assessment framework (HHSAF) proposed by the WHO, which has been included among performance indicators of our region since 2014 [28].

Concerning patient-level data, a HAI prevalence of just over 8% among non COVID-19 patients was identified in this study, which was similar to results of the previous edition of the PPS in Italy (8.03%) and in our region (7.26%), even though patients were slightly older in the current edition [7, 25]. As in the previous edition, the most frequent HAI types were pneumonia, UTIs, and BSIs. In light of the high level of implementation of IPC practices, further research is necessary to evaluate possible explanations for the lack of improvement in HAI prevalence, such as changes in patient case-mix or the impact of the COVID-19 pandemic on HAI transmission.

Several limitations should be considered when interpreting results of this study. First, as participation in the PPS was voluntary, hospitals with higher interest in IPC could be overrepresented. Further, the study mainly focused on public hospitals, with a low participation among private and not-for profit facilities (Table 2). However, as shown in Fig. 1, participation in the PPS accounted for over 50% of total acute-care beds. Second, limitations due to study design apply: we did not attempt to determine causal relationships between variables as point data were collected. Third, data were self-reported, and only patient-level variables were validated. A validation survey according to the ECDC PPS 2022–2023 Validation Protocol Version 4.0 was performed among 50 patients of one facility of the Region, which found a sensitivity and specificity in identifying HAIs of 85.71% and 95.45% respectively. Validation was not performed in regards to the IPCAF questionnaire. Concerning the IPCAF questionnaire in particular, a high degree of understanding of the underlying concepts and definitions was required in order to accurately perform facility assessment, therefore we cannot exclude that certain elements may have been misunderstood or that self-reported results may have been distorted by a degree of reporting bias. Finally, as the primary purpose of the IPCAF is self-assessment, and health system structure, policy, and organization could influence implementation levels, we would caution against international comparisons of results.

In conclusion, through this study a baseline assessment of IPC activities in our region was performed, which allowed us to document strengths and areas for improvement. This first assessment will hopefully be useful to define priorities for action and tailored improvement strategies, in particular considering the high prevalence rates for HAI found in our region. Results of this study provide standardized reference data for benchmarking; repeated monitoring and evaluation could help sustain progress over time [19, 29].

## Data Availability

All data collected within the CCM project “Sostegno alla Sorveglianza delle infezioni correlate all’assistenza anche a supporto del PNCAR”, including those reported in this study, are owned by the Italian Ministry of Health. Data will be made available from the corresponding author upon reasonable request.

## Abbreviations

AMR: Antimicrobial resistance
BSI: Bloodstream infections
CCM: Italian Centre for Disease Control
DALY: Disability-adjusted life years
ECDC: European Centre for Disease Prevention and Control
EU: European Union
FTE: Full-time equivalent
GDPR: General data protection regulation
HAI: Healthcare-associated infection
HELICS: Hospitals in Europe Link for Infection Control through Surveillance
HHSAF: Hand hygiene self-assessment framework
IPC: Infection Prevention and Control
IPCAF: Infection Prevention and Control Assessment Framework
IQR: Interquartile range
ISS: Italian National Health Institute
NHSN: National Healthcare Safety Network
PDs: Patient-days
PNCAR: National Action Plan to contrast AMR
PPS: Point Prevalence Survey
UTI: Urinary tract infections
WHO: World Health Organization
